# Root Damage by Insects Reverses the Effects of Elevated Atmospheric CO_2_ on Eucalypt Seedlings

**DOI:** 10.1371/journal.pone.0079479

**Published:** 2013-11-18

**Authors:** Scott N. Johnson, Markus Riegler

**Affiliations:** Hawkesbury Institute for the Environment, University of Western Sydney, Penrith, New South Wales, Australia; DOE Pacific Northwest National Laboratory, United States of America

## Abstract

Predicted increases in atmospheric carbon dioxide (CO_2_) are widely anticipated to increase biomass accumulation by accelerating rates of photosynthesis in many plant taxa. Little, however, is known about how soil-borne plant antagonists might modify the effects of elevated CO_2_ (eCO_2_), with root-feeding insects being particularly understudied. Root damage by insects often reduces rates of photosynthesis by disrupting root function and imposing water deficits. These insects therefore have considerable potential for modifying plant responses to eCO_2_. We investigated how root damage by a soil-dwelling insect (*Xylotrupes gideon australicus*) modified the responses of *Eucalyptus globulus* to eCO_2_. eCO_2_ increased plant height when *E. globulus* were 14 weeks old and continued to do so at an accelerated rate compared to those grown at ambient CO_2_ (aCO_2_). Plants exposed to root-damaging insects showed a rapid decline in growth rates thereafter. In eCO_2_, shoot and root biomass increased by 46 and 35%, respectively, in insect-free plants but these effects were arrested when soil-dwelling insects were present so that plants were the same size as those grown at aCO_2_. Specific leaf mass increased by 29% under eCO_2_, but at eCO_2_ root damage caused it to decline by 16%, similar to values seen in plants at aCO_2_ without root damage. Leaf C:N ratio increased by >30% at eCO_2_ as a consequence of declining leaf N concentrations, but this change was also moderated by soil insects. Soil insects also reduced leaf water content by 9% at eCO_2_, which potentially arose through impaired water uptake by the roots. We hypothesise that this may have impaired photosynthetic activity to the extent that observed plant responses to eCO_2_ no longer occurred. In conclusion, soil-dwelling insects could modify plant responses to eCO_2_ predicted by climate change plant growth models.

## Introduction

### Background and Rationale

Predicted increases in atmospheric carbon dioxide (CO_2_) concentrations are typically expected to increase plant biomass of C_3_ plants by 10–20% and C_4_ plants by 0–10% [1]. Increased rates of photosynthesis in response to elevated CO_2_ (eCO_2_) underpin these increases in plant biomass, but this is only sustainable with improved nitrogen and water use efficiency in the plant [1], although other physiological processes are clearly important (e.g. [2]). Root function plays an important role in nitrogen and water use efficiency [3], and root growth usually increases relative to shoot growth for most plant species under elevated eCO_2_ conditions [4]–[6]. Combined with greater water use efficiency through reduced stomatal conductance, this investment in root growth and changes in root architecture potentially allows plants to sustain higher levels of photosynthesis at eCO_2_ and ultimately accumulate more biomass [7], [8].

While a number of studies address how insect herbivores moderate plant growth responses to eCO_2_, with several reviews [9]–[11] now published, these largely overlook soil-borne pests of plant roots [12], [13]. There is virtually no information about how soil-dwelling insects are affected by eCO_2_ [14] and even less about how this might impact on plant growth responses to eCO_2_. For example, only three studies have examined the effects of eCO_2_ on root herbivores [15]–[17]. Soil-dwelling insects have the capacity to damage roots either through direct herbivory or physical abrasion to the roots as they move around the rhizosphere [18]. Soil-dwelling insects can be particularly damaging to plant physiology since even minor root damage can: (i) decrease nutrient and water uptake, (ii) cause disproportionate resource losses by severing roots, (iii) divert assimilates belowground for root re-growth and (iv) impair photosynthesis by imposing water deficits [18], [19]. This last point may be critical for plant growth responses under eCO_2_ since increased rates of photosynthesis underpin enhanced growth. Root damaging insects might therefore have greater capacity to reduce, negate or even reverse the effects of eCO_2_ than aboveground herbivores. This hypothesis is supported by two meta-analyses which reported contrasting effects of above- and belowground herbivores on photosynthesis rates; the former often accelerated photosynthesis rates, potentially to compensate for loss of photosynthetic tissue [20], whereas soil insect herbivores significantly reduced it [19].

### Eucalypts and Soil-borne Antagonists

Hovenden & Williams [21] report that 11 species of *Eucalyptus* have been studied in the context of eCO_2_ before 2010, and at least eight of these show strong positive responses in terms of growth. *Eucalyptus* therefore represented a model system to test whether the effects of eCO_2_ would be modified when roots were challenged by soil insect herbivores because of this consistently positive response to eCO_2_. Moreover, eucalypts dominate the 164 million ha of forest in Australia [22], and are now the most widely planted hardwood species in the world [23].

Soil-borne pests and pathogens of *Eucalyptus* roots include numerous microbial diseases [24] and nematodes [25], but also a number of soil insect herbivores [26]. These include termites [27], [28], moth larvae [29] and scarab beetles [26], which have the capacity to cause significant losses in nursery production. Some soil-dwelling insects feed on both living roots and decaying organic matter, but even in the latter case they can cause physical damage of roots through their activity in the rhizosphere [18]. Moreover, with the global spread and movement of *Eucalyptus* it is likely that new and exotic soil-dwelling insects may be accidentally introduced to *Eucalyptus* [22].

### Study System

This study was based on predicted atmospheric CO_2_ concentrations for 2050 onwards [30] and used *Eucalyptus globulus* Labill. (Myrtaceae), which is both a dominant eucalypt species in South Eastern Australia and globally the most widely planted hardwood species in temperate regions [31]. To impose insect damage to *E. globulus* roots we used the soil-dwelling larvae of the generalist feeder *Xylotrupes gideon australicus* L. (Coleoptera: Scarabaeidae). *Xylotrupes* spp. are sporadic pests of forestry and horticulture [32], with soil-dwelling larval stages feeding on decaying organic matter [33] and roots [34]. While this species has not been reported in eucalypt plantations, we used this scarab beetle as a model substitute that has the capacity to impose root damage via herbivory and mechanical abrasion. Since secondary metabolites are not readily inducible by herbivory in eucalypts [35], [36], root damage arising from either activity should be functionally similar.

### Aims and Hypotheses

This study aimed to determine the effects of aCO_2_ and eCO_2_ (400 and 600 µmol mol^−1^, respectively) on the growth, biomass accumulation, leaf morphology and primary chemistry of developing *E. globulus* saplings and determine whether brief (14d) exposure to root damage by soil insects moderated these effects. We hypothesised that eCO_2_ would promote plant height, biomass accumulation, increase specific leaf mass and decrease nitrogen concentrations in plant tissue, but each of these effects would be arrested when roots are challenged by root herbivores.

## Materials and Methods

### Growth Conditions and Experimental Design

Six glasshouse chambers, three maintained at ambient CO_2_ (aCO_2_) concentrations of 400 µmol mol^−1^ and the other three at eCO_2_ concentrations of 600 µmol mol^−1^, were used in this study. Glasshouse chambers (3 m×5 m×3 m; width×length×height) with UV transparent plexiglass (6 mm thick) walls and roof were used and naturally lit throughout the experiment. During the experiment, air temperature within each chamber was maintained according to a diurnal cycle, peaking at 25°C and falling to 20°C (±4°C). Humidity was controlled at 60% (±5%). CO_2_ levels were controlled via a monitoring and control system, PlantVisorPRO (Carel Industries, Padova, Italy). Briefly, CO_2_ levels within each chamber were monitored by a CO_2_ probe (GMP222, Vaisala, Vantaa, Finland), with CO_2_ (food grade, AirLiquide, Australia) injected from pressurized cylinders through solenoid valves. Before entering a chamber, CO_2_ was passed through a Purafils column to eliminate possible ethylene contamination. *Eucalyptus globulus* plants were grown from seed (CSIRO Australian Tree Seed Centre, Seedlot number 18673) in commercial potting mixture (Plugger Custom, Debco Pty Ltd., NSW, Australia). Once established, 96 viable and similar sized plants were transferred into square pots (90 mm×90 mm×180 mm, width×length×height) filled with c. 750 g of air dried soil sieved <4 mm. The soil was loamy-sand with low (0.7%) organic matter (see [37] for full soil properties). Plants were then randomly assigned to each of the six climate chambers (16 in each). Plants were watered daily (c. 300 mL) to maintain soil water content at around 10% (verified with two rod soil moisture probe, Hydrosense, Campbell Scientific, Australia) and supplemented monthly with liquid fertilizer (1.6 g L^−1^ Aquasol, N:P:K 23∶4:18). *Xylotrupes gideon australicus* larvae were maintained in culture at 20°C ±5°C in a mixture of pine bark mulch (Richgro, Jandakot, WA, Australia) and soil (as above) until required.

### Experimental Procedure

Once plants were 13 weeks old, plant height was recorded at weekly intervals until the end of the experiment. After a further week, 48 of the *X. gideon* larvae (first instar) which had been starved for 48 hr were weighed and individually applied to half of the plants (assigned at random) in each of the chambers. Detailed information about likely densities of soil-dwelling insects in eucalypt systems is lacking, but our previous research indicated grass-feeding scarab densities in eucalypt plantations would approximate this [38]. Larvae were placed in an excavated hole at the corner of the pot, which was then backfilled with soil. After two weeks, larvae were removed from the pots by gentle excavation of the soil and re-weighed. Similar soil excavation was performed on plants without larvae to replicate any effects of this disturbance. Plants were left for a further week before carefully removing from the soil, whereupon they were weighed and separated into stems, leaves and roots. All detached root material in the pots was also collected and included in the root mass evaluation to establish the extent of root herbivory as opposed to mechanical damage caused by larval movement. To calculate specific leaf mass, a single leaf from the middle of the plant was weighed, measured for leaf area, snap frozen in liquid nitrogen, freeze dried and re-weighed. All remaining plant tissue was snap frozen in liquid nitrogen and stored at −20°C. All plant material was subsequently freeze dried, weighed and milled to analyse carbon (C) and nitrogen (N) concentrations using a LECO TruSpec® CHN analyser.

### Statistical Analysis

All plant responses were analysed with two-way analysis of variance (ANOVA) tests, with chamber replicate (three chambers at each CO_2_ regime) included as a block term to avoid issues of pseudo-replication of CO_2_ treatment. CO_2_ and insect presence, and an interaction of the two, were the two fixed effects. Differences between individual treatment combinations were determined with least square difference tests. In the case of plant height, separate ANOVAs for each time point were conducted since repeated measures ANOVA was inappropriate due to insects only being present during three of five points that height was measured (i.e. these were not fully repeated events). Final mass of insects was analysed with a one-way ANOVA with CO_2_ as the fixed effect, chamber included as the block term and initial mass included as a covariate. Unless otherwise stated in Table 1 all analysis was conducted on untransformed data using Genstat (version 15, VSN International, UK). Transformations were chosen to give residual diagnostic plots which best fitted a normal distribution and showed least heteroscedasticity.

**Table 1:** Results of ANOVA tests of plant responses to aCO_2_ and eCO_2_ and root damaging insects (RD) relating to Figs. 1–4.

Plant response	Figure	Factors					
		CO_2_		RD		CO_2_ × RD	
		F_1,4_	*P*	F_1,88_	*P*	F_1,88_	*P*
Plant height –13 weeks	1	1.53	0.284	0.76	0.386^1^	0.09	0.762
Plant height –14 weeks		**7.48**	**0.050**	0.89	0.348^1^	0.06	0.814
Plant height –15 weeks		5.54	0.078	2.76	0.100	0.43	0.541
Plant height –16 weeks		**10.06**	**0.034**	**18.30**	**<0.001**	**5.70**	**0.019**
Plant height –17 weeks		**13.25**	**0.022**	**22.52**	**<0.001**	2.67	0.106
Plant mass(total)	2	**83.20**	**<0.001**	**12.83**	**<0.001**	0.94	0.335
Shoot mass		**107.45**	**<0.001**	**12.63**	**<0.001**	1.00	0.321
Root mass		**28.87**	**0.006**	**6.83**	**0.011**	0.07	0.789
Shoot : root		3.19	0.148	1.28	0.261	1.81	0.182
Specific leafmass	3A	**12.56**	**0.024**	**6.28**	**0.014**	2.68	0.105
Leaf watercontent	3B	0.10	0.762	**5.69**	**0.019**	0.38	0.541
Leaf C:N	4	**14.27**	**0.019**	0.01	0.942	**5.24**	**0.024**

Significant effects (*P*<0.05) indicated in bold.

1 Measurements taken on plants assigned for root herbivore treatment prior to inoculation with insects. Statistical tests indicating no priori difference between plants assigned for inoculation.

## Results

All insects were recovered alive and roots showed considerable herbivory and root detachment. Including detached root tissue, total root mass was c. 15% lower in pots containing larvae indicating that root tissue had been consumed by insects. While absolute root consumption and removal could not be determined exactly on the basis of differences between infested and control (i.e. insect-free) plants, this differences was similar under aCO_2_ and eCO_2_ (910 and 1130 mg in dry mass, respectively) and suggested root consumption was similar. The final body mass of beetle larvae was largely unaffected by eCO_2_ (F_1,3_ = 0.57, *P = *0.613; data not shown).

### Plant Growth and Biomass Accumulation

**Figure 1:**
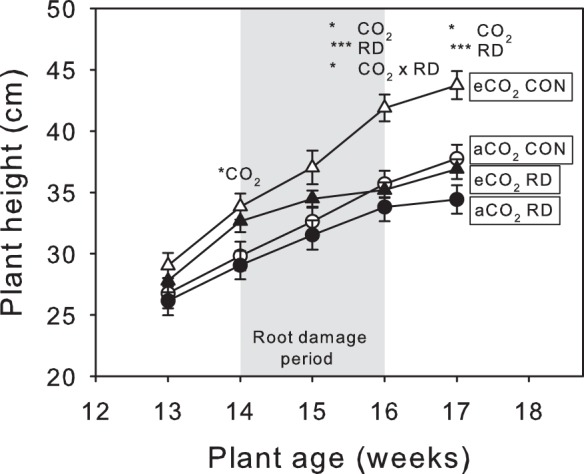
Plant height as affected by root damage and CO_2_. Height immediately preceding, during (shaded grey) and after root damage (RD) on plants under aCO_2_ (circles) and eCO_2_ (triangles). Open symbols are control plants (CON), closed symbols are plants with insect root damage (RD). Mean values ± S.E. shown, N = 24. Statistical significance of treatments indicated *(*P*<0.05), **(*P*<0.01) and *** (*P*<0.001).

Plant height was significantly greater for plants grown at eCO_2_ than those at aCO_2_ by the time plants were 14 weeks old, which was also the case at 16 and 17 weeks (Fig. 1; Table 1). Application of insects had no impact for the first week, but caused a sharp decline in growth after 14 days which persisted after their removal (Fig. 1; Table 1).

**Figure 2:**
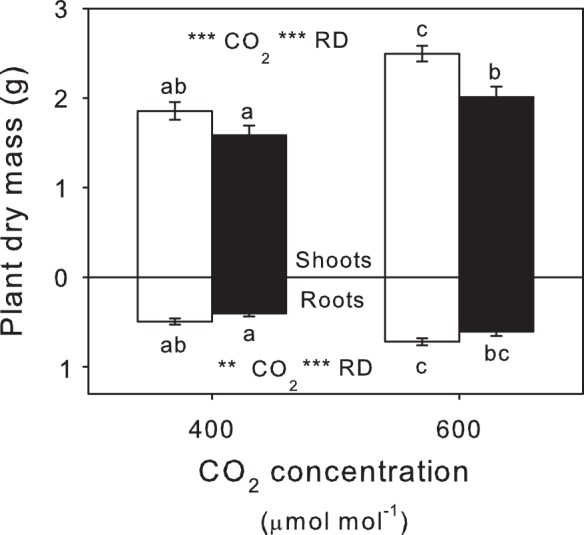
Plant biomass (dry) as affected by root damage and CO_2_. Shoot and root mass of plants at aCO_2_ and eCO_2_ without (open bars) and with (closed bars) root damage (RD). Mean values ± S.E. shown, N = 24. Statistical significance of treatments indicated **(*P*<0.01) and *** (*P*<0.001) with lowercase superscript letters indicating significant differences (*P*<0.05) between treatments.

eCO_2_ promoted growth of both shoots and roots, resulting in bigger plants overall (Fig. 2; Table 1). On plants without insects, eCO_2_ caused a 46% and 35% increase in root and shoot biomass, respectively, though there was no statistically significant change in the shoot:root ratio (Table 1). Exposure to insects had the opposite effect to eCO_2_, reducing both root and shoot mass. This arrested the positive effects of CO_2_ and left plants with insects under eCO_2_ effectively the same size as those grown at aCO_2_ without root damage (Fig. 1). Under aCO_2_, application of insects resulted in a in a 14.7% reduction in shoot biomass which increased to a 19.4% reduction under eCO_2_. Root loss due to insect damage was 18.42% and 15.7% under aCO_2_ and eCO_2_, respectively.

### Leaf Traits

**Figure 3:**
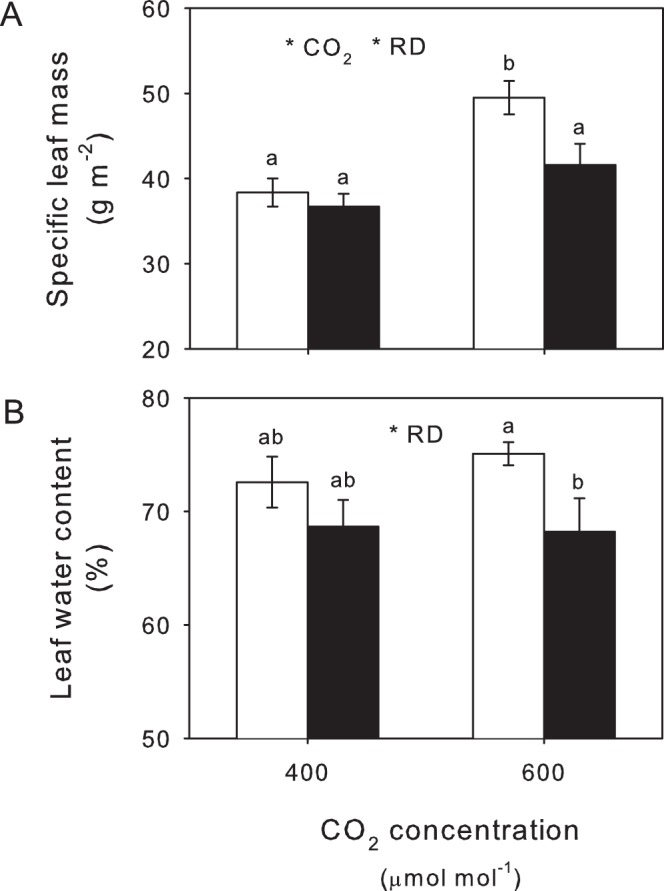
Leaf traits as affected by CO_2_ and root damage. Effects of aCO_2_ and eCO_2_ on (A) specific leaf mass and (B) leaf water content (%) with (closed bars) and without (open bars) root damage (RD). Mean values ± S.E. shown, N = 24. Statistical significance of treatments indicated *(*P*<0.05) with lowercase superscript letters indicating significant differences (*P*<0.05) between treatments.

Specific leaf mass was positively affected by eCO_2_ (Fig. 3A; Table 1), whereas root damage caused this to decline (Table 1). Moreover, plants at eCO_2_ with root herbivores had specific leaf mass values similar to those at aCO_2_ (Fig. 3A). While the interaction between CO_2_ and insect presence was not statistically significant at a 95% confidence interval (*P* = 0.105; Table 1), insects appeared to be having a more negative impact on specific leaf mass at eCO_2_. Root damage reduced leaf water concentrations overall (Table 1), but this difference largely occurred under eCO_2_ (Fig. 3B).

### Primary Chemistry

**Figure 4:**
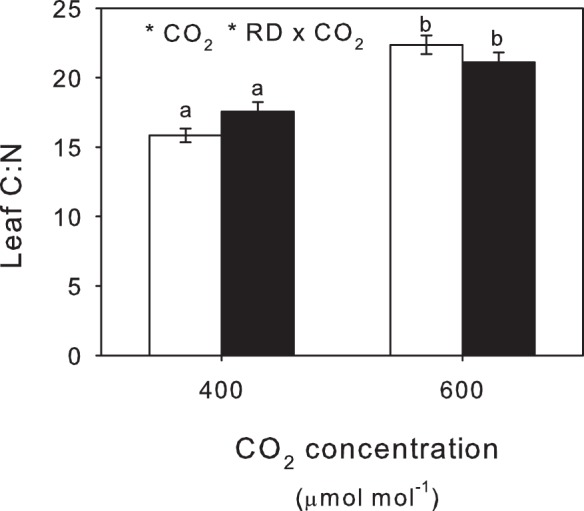
Leaf C:N ratios. Effects of aCO_2_ and eCO_2_ on leaf C:N from plants without (open bars)and with root damage (RD). Mean values ± S.E. shown, N = 24. Statistical significance of treatments indicated *(*P*<0.05) with lowercase superscript letters indicating significant differences (*P*<0.05) between treatments.

Leaf C:N ratio rose in plants grown under eCO_2_ (Fig. 4; Table 1), driven largely by a decline in leaf N concentrations under eCO_2_ (Table 2). The significant interaction between eCO_2_ and root damage (Table 2) reflected the opposing effects of root damage on leaf N concentrations, causing a small reduction and increase at aCO_2_ and eCO_2_, respectively. Root damage had no impact on root C and N concentrations and similarly these remained largely unchanged by eCO_2_ (Table 2).

**Table 2:** Carbon and nitrogen concentrations of shoots and roots of plants grown at aCO_2_ (400 µmol mol^−1^) and eCO_2_ (600 µmol mol^−1^) with and without root damaging insects (RD).

CO_2_ concentration(µmol mol^−1^)	Herbivory	Leaf N (mg g^−1^)	Leaf C^1^ (mg g^−1^)	Root N (mg g^−1^)	Root C (mg g^−1^)
400	Absent	^a^ 30.7±6.3	477.0±97.3	12.8±2.6	470.7±96.1
	Present	^a^ 28.7±5.9	479.1±97.8	12.6±2.6	473.6±96.7
600	Absent	^b^ 22.0±4.5	480.0±98.0	12.4±2.5	467.9±95.5
	Present	^b^ 23.4±4.8	480.6±98.1	13.3±2.7	470.3±96.0
CO_2_		**F_1,4_ = 12.02**	F_1,4_ = 0.15	F_1,4_ = 0.30	F_1,4_ = 0.18
		***P*** ** = 0.026**	*P* = 0.716	*P* = 0.611	*P* = 0.697
RD		F_1,88_ = 0.15	F_1,88_ = 0.07	F_1,88_ = 0.19	F_1,88_ = 1.37
		*P* = 0.701	*P* = 0.790	*P* = 0.667	*P* = 0.244
CO_2_ × RD		**F_1,88_ = 4.82**	F_1,88_ = 0.11	F_1,88_ = 2.32	F_1,88_ = 0.01
		***P*** ** = 0.031**	*P* = 0.746	*P* = 0.132	*P* = 0.907

Mean values ± S.E shown, N = 24. Significant effects indicated in bold. Lowercase superscript letters indicates significant differences (*P*<0.05) between treatments.

1 Arcsine square root transformation applied.

## Discussion

This study set out to establish whether root damage by soil-dwelling insects modified the response of *E. globulus* seedlings to eCO_2_. The findings suggest that this is the case, with root damage substantially reducing biomass accumulation by *E. globulus* under eCO_2_ and effectively reversing effects of eCO_2_ on specific leaf mass.

### Plant Growth and Leaf Traits

In agreement with the meta-analysis by Zvereva & Kozlov [19], we found that insect herbivory or damage substantially reduced aboveground biomass by 19.4% and 14.7% at aCO_2_ and eCO_2_, respectively; both are similar values to their global prediction of 16.3%. The fact that enhanced plant growth was not achieved at eCO_2_ in the presence of root damaging insects suggests that plants were likely unable to accelerate or maintain rates of photosynthesis to capitalise on eCO_2_ conditions. The link between increased eucalypt growth under eCO_2_ and higher rates of photosynthesis is well established [39], so it is possible that root damage by soil insects imposed water deficits (consistent with the reported lower foliar water %) which limited photosynthetic activity.

Attendant changes in leaf traits also support this hypothesis, with insects reducing specific leaf mass which we reported as being increased under eCO_2_, in common with at least seven other eucalypt species [39]–[45]. Specific leaf mass, and implicitly leaf thickness, commonly increase under eCO_2_ which also renders leaves less palatable for leaf herbivores and reduces their performance [9], including eucalypts [44], [45]. Root herbivores frequently affect aboveground herbivores through plant-mediated mechanisms under aCO_2_ [46], so this raises the potential for root damaging insects to alter predicted effects of eCO_2_ on foliar herbivores. In this particular system, this could leave *E. globulus* more susceptible to defoliators under eCO_2_ when roots were under attack.

### Leaf Chemistry

This study also found an increase in leaf C:N under eCO_2_ which is widely reported for many plant species [10], [47], and is normally attributed to a dilution effect as plants increase allocation to non-structural carbohydrates. In addition, higher leaf C:N can arise as plants increase N use efficiency and reduce allocation to Rubisco under eCO_2_ [1]. In common with *E. saligna* and *E. sideroylon* [39], we found that eCO_2_ reduced leaf N concentrations, which is consistent with plants allocating less N to Rubisco. The only other study, to our knowledge, to examine the effects of eCO_2_ on *E. globulus* also found changes in primary chemistry [48], though these were more modest, possibly due to different experimental conditions.

The effects of root damage on leaf chemistry were more complex, with a significant interactive effect of CO_2_ and insect presence. This arose because insects affected leaf N in opposing ways depending on CO_2_; marginally reducing leaf N concentrations under aCO_2_, but increasing it relative to control plants under eCO_2_ (with corresponding increases and decreases in leaf C:N, respectively). We hypothesise that root damage by insects could impair root uptake of N, resulting in a decrease in leaf N at aCO_2_ (for example, root herbivory has been reported to reduce N uptake by up to 30%; e.g. [49]). At eCO_2_, however, root damage could have reduced nitrogen use efficiency to the extent that plants could not re-allocate N (i.e. reduce foliar levels) to the same extent as in plants without root damaging insects.

### Eucalypts and Soil-borne Antagonists in Future Climates

Most attention concerning soil-borne antagonists of eucalypts focus on plant pathogens [24], but several soil insect herbivores clearly attack eucalypt roots [26]. These are less conspicuous and currently pose less of a threat than aboveground herbivores (the latter are reviewed by [22], [50]). However, Wilcken *et al*. [28] reported that up to 70% of nursery eucalypt seedlings were killed by root herbivores, clearly demonstrating their destructive potential. Moreover, root-feeding insect herbivores are often highly invasive, with exotic species becoming significant pests of forest systems, as is the case in North America [51], so new pests could become apparent [22]. In particular, the results of the present study indicate that beneficial effects of eCO_2_ on eucalypt performance would be negated by root damaging insects in nursery aged plants.

In the current study, applying root damaging insects and eCO_2_ in a controlled manner necessitated glasshouse experiments. Glasshouse studies do not always reflect plant responses seen under field conditions [3], so our conclusions have to be viewed in this context. Having said this, early results from field based whole tree chambers [37] suggest *E. globulus* sapling growth responses to eCO_2_ in the field are consistent with the findings reported here (D. Ellsworth, pers. comm.) and elsewhere ( [21] and references therein). Another constraint was the use of pots to conduct this experiment, which sometimes affect growth responses [52] and may have slightly increased root damage by constraining herbivores. We minimised these effects by using a free draining soil (which minimises the chances of hypoxic conditions recommended in [52]). Also, root herbivores generally show restricted movement and usually remain associated with the root system when resources are adequate [53] so this probably was not a major issue for the brief period of root damage we applied.

## Conclusions

This study has illustrated the potential for soil-dwelling insect herbivores to arrest or reverse the effects of eCO_2_ on plant physiology and biomass accumulation. Our results suggest that root damage by these insects (arising through herbivory and mechanical attrition) impaired water uptake which may have curtailed photosynthesis activity and limited the plant’s capacity for biomass accumulation at eCO_2_. The recent revelation that the majority of root herbivores reduce plant photosynthesis rates (by an average of 12%), whereas defoliators do the opposite [19], suggests that belowground herbivores might have more scope for modifying plant responses to eCO_2_ than aboveground herbivores. The present study provides some empirical basis for developing and testing hypotheses about how root damage by soil-dwelling insects may moderate plant responses to eCO_2_.
